# Prosthetic Meshes for Repair of Hernia and Pelvic Organ Prolapse: Comparison of Biomechanical Properties

**DOI:** 10.3390/ma8052794

**Published:** 2015-05-22

**Authors:** Manfred M. Maurer, Barbara Röhrnbauer, Andrew Feola, Jan Deprest, Edoardo Mazza

**Affiliations:** 1 Institute of Mechanical Systems, ETH Zurich, Leonhardstrasse 21, Zurich 8092, Switzerland; E-Mails: barbara.roehrnbauer@gmx.de (B.R.); mazza@imes.mavt.ethz.ch (E.M.); 2 Center for Surgical Technologies, Faculty of Medicine, Universitair Ziekenhuis “Gasthuisberg” Leuven, Katholieke Universiteit Leuven, Leuven 3000, Belgium; E-Mails: feolaaj12@gmail.com (A.F.); Jan.Deprest@uzleuven.be (J.D.); 3 Empa—Swiss Federal Laboratories for Materials Science and Technology, Überlandstrasse 129, Dübendorf 8600, Switzerland

**Keywords:** mechanical biocompatibility, mesh prostheses, POP meshes, hernia meshes

## Abstract

This study aims to compare the mechanical behavior of synthetic meshes used for pelvic organ prolapse (POP) and hernia repair. The analysis is based on a comprehensive experimental protocol, which included uniaxial and biaxial tension, cyclic loading and testing of meshes in dry conditions and embedded into an elastomer matrix. Implants are grouped as POP or hernia meshes, as indicated by the manufacturer, and their stiffness in different loading configurations, area density and porosity are compared. Hernia meshes might be expected to be stiffer, since they are implanted into a stiffer tissue (abdominal wall) than POP meshes (vaginal wall). Contrary to this, hernia meshes have a generally lower secant stiffness than POP meshes. For example, DynaMesh PRS, a POP mesh, is up to two orders of magnitude stiffer in all tested configurations than DynaMesh ENDOLAP, a hernia mesh. Additionally, lighter, large pore implants might be expected to be more compliant, which was shown to be generally not true. In particular, Restorelle, the lightest mesh with the largest pores, is less compliant in the tested configurations than Surgipro, the heaviest, small-pore implant. Our study raises the question of defining a meaningful design target for meshes in terms of mechanical biocompatibility.

## 1. Introduction

Mechanical biocompatibility of prosthetic meshes for hernia and pelvic organ prolapse (POP) is related to the ability of implants to display a mechanical behavior compatible with its function and favoring its integration into the surrounding native tissue [1,2,3,4,5,6]. This approach for implant assessment has received increased attention in recent years. While initial investigations focused on the ability of a mesh to provide sufficient strength and resistance to maximum loads [7,8,9,10,11,12], it recently became clear that the deformation behavior in a physiological range, also called “comfort zone”, is of major importance [13,14]. A mismatch of mechanical properties of the implants compared to native tissue has been associated with clinical complications [15,16,17,18,19], although none of these works explicitly link mechanical properties with clinical outcome. It has recently been suggested that meshes designed to mimic the biomechanical properties of the area of application are advantageous [2,14,20]. These investigations are further motivated by an FDA safety communication [21] pointing at risks associated with existing prosthetic meshes and corresponding surgery procedures for repair of POP.

A wealth of studies has been conducted analyzing either hernia or POP meshes (see [3,22] for an extensive literature overview). However, little work was performed to compare the mechanical response of these two groups, which may shed light onto the prevalent clinical complications. The mechanical environment and loading conditions these implants are exposed to differ significantly between the abdominal wall and pelvic floor. Physiological loads in terms of membrane tension were calculated based on Laplace’s law to be around 0.035 N/mm in the pelvic region and 0.136 N/mm at the abdominal wall at rest, but can be orders of magnitude higher at increased intra-abdominal pressures [5,7,23]. This gives an indication of the range of load at which mesh implants should work best in supporting and mimicking native tissue, thus ensuring mechanical biocompatibility.

Based on the experimental study presented in [22], the data analysis in the present investigation is extended to compare the mechanical properties of hernia and POP mesh implants with respect to physiological loading conditions.

## 2. Experimental Section

Nine mesh implants were investigated. They were grouped into hernia (n = 5) and POP (n = 4) implants based on the manufacturer’s information, available on their respective websites and analyzed accordingly. All products are described in Table 1.

The mechanical testing procedure has been previously described in detail [22]. In short, each mesh type was tested in eight different configurations: 2 (uniaxial tension or biaxial tension) × 2 (dry or embedded) × 2 (0° or 90° direction).

**Table 1:** List of mesh types used for the present investigation, with their weight classified as ultralight, light or standard according to [24]. Principal directions of testing are marked in red. Scale bar (lower right): 5 mm. Their clinical application is listed as pelvic organ prolapse (POP) or hernia repair, as specified by the manufacturer.

Image	Mesh	Application	Material, Weight
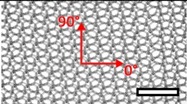	Bard™ Mesh Marlex (BM)	Hernia	Polypropylene, standard
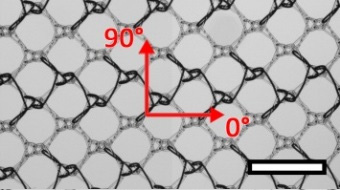	DynaMesh^®^ ENDOLAP (DM)	Hernia	PVDF (polyvinylidene fluoride), standard
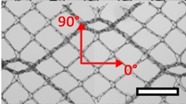	Ethicon Physiomesh^®^ (PM)	Hernia	Polypropylene, ultralight
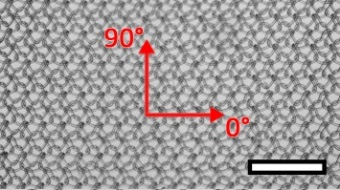	Surgipro™ Polypropylene Monofilament Mesh (SPMM)	Hernia	Polypropylene, standard
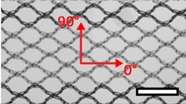	Ethicon Ultrapro™ (UP)	Hernia	Polypropylene, light
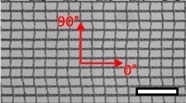	DynaMesh^®^ PRS (DMPRS)	POP	PVDF, standard
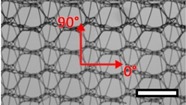	Gynecare PROLIFT™ (PE)	POP	Polypropylene, ultralight
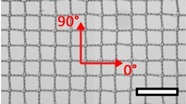	Coloplast Restorelle™ (Rest)	POP	Polypropylene, ultralight
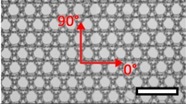	Parietex Ugytex^®^ (UT)	POP	Polypropylene, light

These test configurations represent the *in vivo* loading and environmental conditions of the mesh implants. Long, narrow strips of meshes used in “line-type” suspensions are mainly loaded in uniaxial tension, whereas wider sheets, such as for hernia repair, are typically subjected to multiaxial tension states. Our earlier study examined the anisotropic behavior of these meshes along two perpendicular directions following the main knitting patterns. However, here, the focus is on the stiffer of the two directions on a per mesh basis. A dry mesh is tested as delivered, whereas embedded infers a specimen being embedded into a soft elastomer matrix (Young’s modulus 0.0276 N/mm^2^ [25]), mimicking *in vivo*, ingrown conditions.

Experiments with uniaxial tension and biaxial tension (realized as uniaxial strain test, also called “strip biaxial”; [22,26]) were performed on the same tensile test machine. In the uniaxial strain test, lateral contraction of the specimen is constrained, leading to stresses in the direction perpendicular to the loading axis, thus subjecting the sample to a biaxial state of tension. Test piece dimensions were selected to generate a free area of 30 mm × 15 mm (uniaxial) and 50 mm × 15 mm (biaxial). Each specimen was loaded to a maximum of 30% nominal strain (loading rate ~10^−3^s^−1^) and unloaded back to a pre-force threshold of 0.01 N for 10 cycles.

Deformation analysis was performed in an optical, non-contact procedure in the center of the specimen, allowing for extraction of local strains (*ε_loc_*) as the result of an image analysis algorithm, thus avoiding edge and clamp effects at the specimen boundaries. Force measurements at the clamps were converted to nominal membrane tension (*M_t_* (N/mm)) by dividing by the undeformed width of the sample. For a detailed description of the loading protocol and data extraction, refer to [22].

The resulting *M_t_*–*ε_loc_* curves of each of the 8 specimens of each type, as well as the area density and porosity measurements [27,28] form the basis for the analysis and comparison of mesh groups. Dry mesh samples of known dimensions were weighted before mechanical testing using a high-resolution balance, and their area density was calculated as weight per area (kg/m^2^). Porosity is determined as the ratio of open area to the total area, including filaments of one undeformed unit cell of the knitting pattern [27,28].

**Figure 1:**
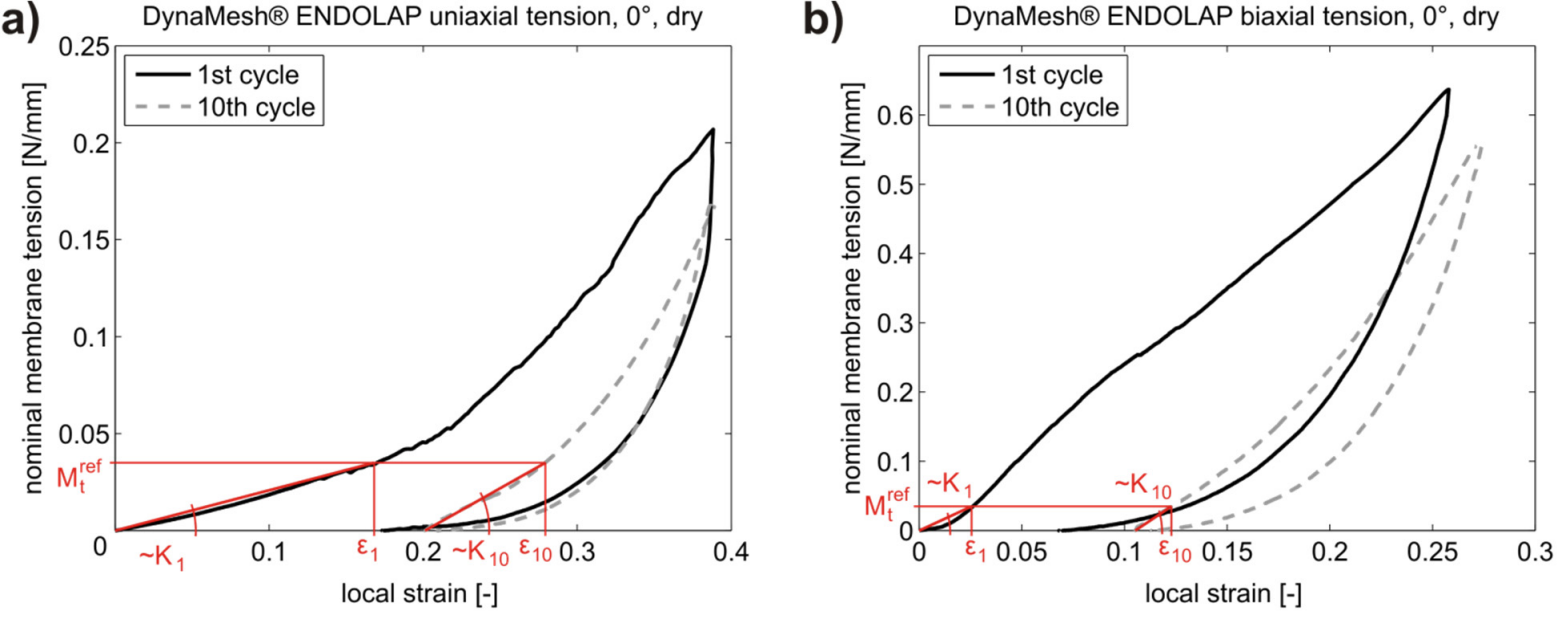
Exemplary tension-strain curves for DynaMesh ENDOLAP (DM) for the illustration of secant stiffness determination. (**a**) Uniaxial tension in the 0° direction, dry mesh, loading and unloading in 1st and 10th cycle; (**b**) Biaxial tension in the 0° direction, dry mesh, loading and unloading in 1st and 10th cycle. Secant stiffness in the 1st and 10th cycle is shown for both.

From the *M_t_*–*ε_loc_*, curves the secant stiffness *K* (N/mm) in the stiffer direction at the reference membrane tension $M_{t}^{ref}=0.035\;\text{N}/\text{mm}$ (for hernia, as well as POP meshes) was extracted, for both the 1st and 10th cycle (see Figure 1). It is defined as:

(1) $$K=\frac{M_{t}^{ref}}{∆\varepsilon }$$

where Δɛ is the difference of local strain at the reference membrane tension $M_{t}^{ref}$ and at the beginning of the current cycle.

The specific value of *M_t_* was chosen as a load representative of the membrane tension in the pelvic region under physiological intra-abdominal pressure (IAP) at rest [23]. Each mesh is thus characterized by 10 parameters, *i.e.*, a secant stiffness value for each of the tested configurations (uniaxial and biaxial tension, dry and embedded) in 1st and 10th cycle, as well as area density and porosity.

The implants are grouped into POP and hernia meshes as indicated on their official product insert. Each parameter is shown in a bar graph, as well as standard box plots in order to visualize the differences between the two groups. To determine the statistical significance, the Wilcoxon rank sum test (equivalent to the Mann–Whitney U-test) is applied for each parameter.

## 3. Results and Discussion

### 3.1. Results

**Figure 2:**
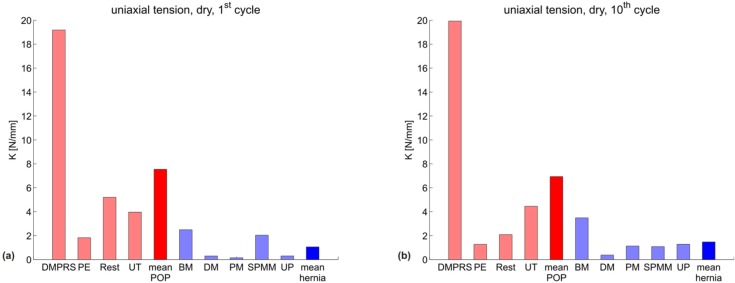

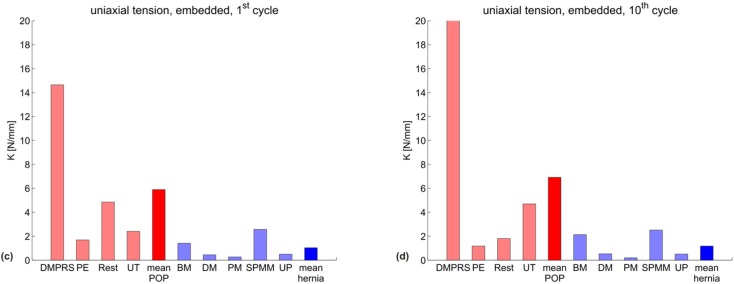
Uniaxial secant stiffness *K_uni_* (N/mm) for all meshes in four configurations: (**a**) dry mesh, first cycle; (**b**) dry mesh, 10th cycle; (**c**) embedded mesh, first cycle; (**d**) embedded mesh, 10th cycle. POP meshes are shown in red; hernia meshes in blue. The mean of each group is plotted darker.

**Figure 3:**
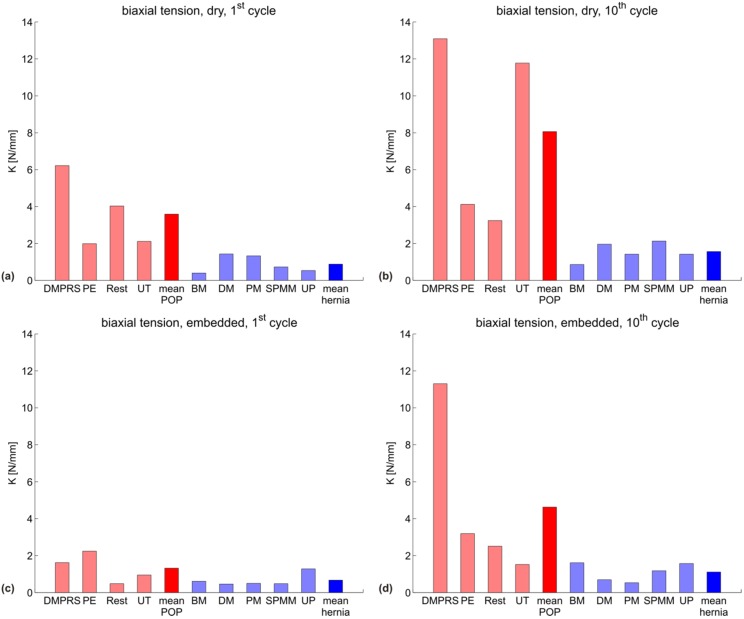
Biaxial secant stiffness *K_bi_* (N/mm) for all meshes in four configurations: (**a**) dry mesh, first cycle; (**b**) dry mesh, 10th cycle; (**c**) embedded mesh, first cycle; (**d**) embedded mesh, 10th cycle. POP meshes are shown in red; hernia meshes in blue. The mean of each group is plotted darker.

Figure 2 shows the uniaxial secant stiffness *K_uni_* (N/mm) of each specimen grouped according to the manufacturer indication into POP (red) and hernia (blue) meshes, with the mean of each group shown in darker red and blue, respectively. The specific testing conditions (dry/embedded and first/10th cycle) are indicated in each subgraph. Figure 3 represents the corresponding biaxial secant stiffness *K_bi_* (N/mm). The respective stiffness values for each configuration are reported in Table 2 (POP meshes) and Table 3 (hernia meshes).

**Table 2:** Numerical values of stiffness for all POP meshes and all tested configurations.

Testing configuration	*K* (N/mm) POP meshes				
	DMPRS	PE	Restorelle	UT	mean
uniaxial, dry, 1st cycle	19.2	1.8	5.2	4.0	7.5
uniaxial, dry, 10th cycle	19.9	1.3	2.1	4.5	6.9
uniaxial, embedded, 1st cycle	14.6	1.7	4.8	2.4	5.9
uniaxial, embedded, 10th cycle	20.0	1.2	1.8	4.7	6.9
biaxial, dry, 1st cycle	6.2	2.0	4.0	2.1	3.6
biaxial, dry, 10th cycle	13.1	4.1	3.2	11.8	8.1
biaxial, embedded, 1st cycle	1.6	2.2	0.5	0.9	1.3
biaxial, embedded, 10th cycle	11.3	3.2	2.5	1.5	4.6

**Table 3:** Numerical values of stiffness for all hernia meshes and all tested configurations.

Testing configuration	*K* (N/mm) hernia meshes					
	BM	DM	PM	SPMM	UP	mean
uniaxial, dry, 1st cycle	2.5	0.3	0.2	2.0	0.3	1.1
uniaxial, dry, 10th cycle	3.5	0.4	1.1	1.1	1.3	1.5
uniaxial, embedded, 1st cycle	1.4	0.5	0.3	2.6	0.5	1.0
uniaxial, embedded, 10th cycle	2.1	0.5	0.2	2.5	0.5	1.2
biaxial, dry, 1st cycle	0.4	1.4	1.3	0.7	0.5	0.9
biaxial, dry, 10th cycle	0.9	2.0	1.4	2.1	1.4	1.6
biaxial, embedded, 1st cycle	0.6	0.5	0.5	0.5	1.3	0.7
biaxial, embedded, 10th cycle	1.6	0.7	0.5	1.2	1.6	1.1

**Figure 4:**
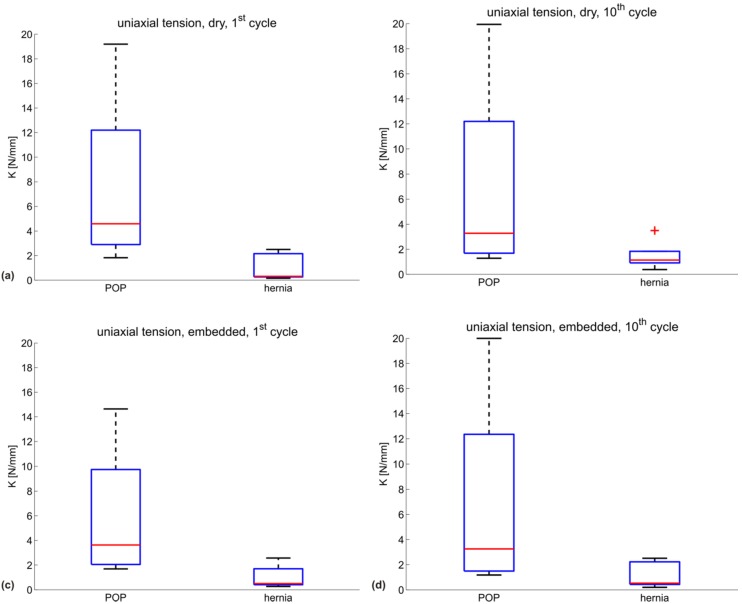
Box plots for uniaxial secant stiffness *K_uni_* (N/mm) for all meshes in four configurations: (**a**) dry mesh, first cycle; (**b**) dry mesh, 10th cycle; (**c**) embedded mesh, first cycle; (**d**) embedded mesh, 10th cycle. The red line marks the median of the group; the box represents the 25th and 75th percentile; the extended whiskers the most extreme data points. The + sign indicates an outlier.

**Figure 5:**
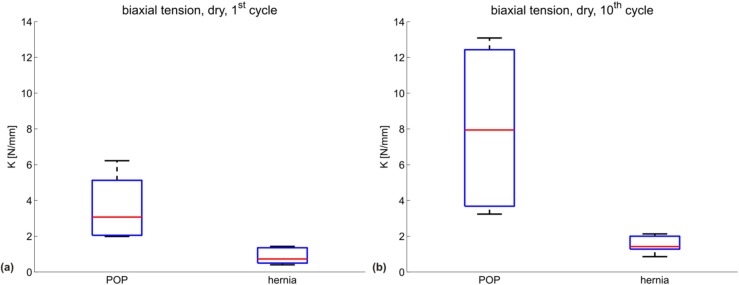

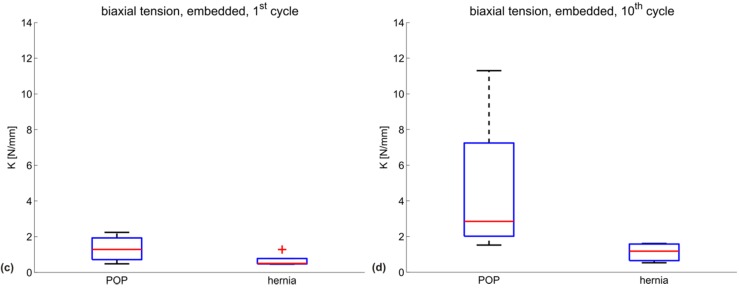
Box plots for biaxial secant stiffness *K_bi_* (N/mm) or all meshes in four configurations: (**a**) dry mesh, first cycle; (**b**) dry mesh, 10th cycle; (**c**) embedded mesh, first cycle; (**d**) embedded mesh, 10th cycle. The red line marks the median of the group; the box represents the 25th and 75th percentile; the extended whiskers the most extreme data points. The + sign indicates an outlier.

Figure 4 and Figure 5 depict the summarizing box plots for the two groups, for uniaxial and biaxial stiffness in each configuration.

The variability for the POP group is very large for all parameters, thus affecting the statistical significance of the differences observed. The Wilcoxon rank sum test indicates a statistically-significant difference between the POP and hernia groups for the biaxial stiffness in the dry condition, both at the first and 10th cycle (*p* = 0.016 for both); see Figure 4a,b and Figure 5a,b. The POP meshes were four- or five-fold stiffer than the hernia meshes in the first cycle and 10th cycle, respectively.

When comparing the mean and median stiffness for all configurations, POP implants are overall less compliant than hernia implants. Since the abdominal wall is known to be stiffer than vaginal tissue [9,29] and if mechanical biocompatibility were mainly dependent on similar properties to the implant area, one would expect a more compliant design for implants for POP compared to hernia. Embedding a mesh into a polymer matrix, thus reflecting the interaction with native tissue, affects the mechanical response of the implants. The differences between the groups are still evident also for this case (see Figure 2, Figure 3 and Figure 4 and Figure 5c,d).

**Figure 6:**
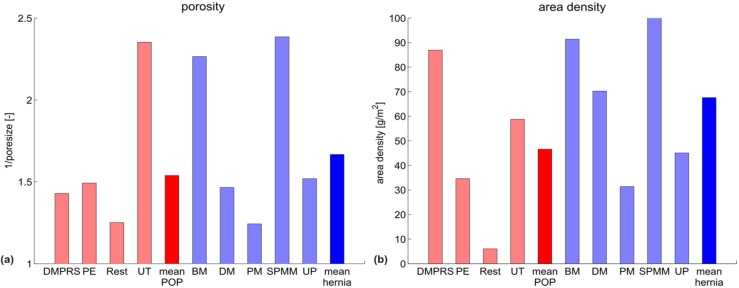
(**a**) Porosity and (**b**) area density of all meshes. POP meshes are shown in red, hernia meshes in blue, with their respective mean values plotted darker. Porosity is shown as the inverse of pore size, *i.e.*, higher values represent smaller pores.

**Figure 7:**
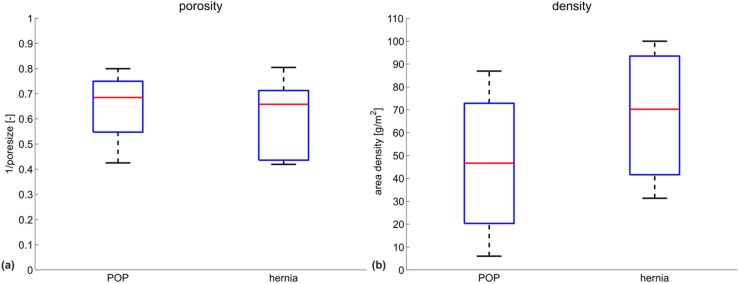
Box plots for (**a**) porosity and (**b**) area density of all meshes. The red line marks the median of the group; the box represents the 25th and 75th percentile; the extended whiskers the most extreme data points. Porosity is shown as the inverse of pore size, *i.e.*, higher values represent smaller pores.

High density and small pores are often linked to high stiffness in prosthetic meshes [30,31]. When comparing the POP and hernia groups, no statistically-significant difference can be found in these parameters (see Figure 6 and Figure 7). However, tendencies can be seen with the hernia meshes being heavier (and similarly porous), while still being in general more compliant.

### 3.2. Discussion

Better clinical outcome might be expected from meshes designed to mimic the physiologically-relevant deformation behavior of the underlying native tissue, thus ensuring mechanical biocompatibility. This entails a meaningful stiffness reference target for mesh design. However, the physiological loading configuration, as well as the range of load levels in terms of membrane tension in the abdominal and vaginal wall still remain largely uncertain. While membrane tensions in the abdominal wall are generally higher [7] than in the pelvic region (simply due to geometric reasons, as shown in [23]), increasing the level of membrane tension at which the secant stiffness is evaluated for the hernia meshes to a level of 0.136 N/mm (reported in [23] as a tension at rest in the abdominal wall) only marginally increases their stiffness and does not change the trends reported in Figure 2, Figure 3, Figure 4, Figure 5, Figure 6 and Figure 7.

The range of stiffness values for native tissue reported in the literature shows large variation and is mostly based on uniaxial tensile tests, while the predominant loading state *in vivo* is biaxial. Song *et al.* [32] report a Young’s modulus of 0.042 N/mm^2^ and 0.0225 N/mm^2^ in the transverse and sagittal plane, respectively, for human abdominal wall during *in vivo* insufflation, which would translate to membrane stiffness values of 1.26 N/mm and 0.675 N/mm, respectively, multiplying by the reported thickness of around 30 mm [32]. Analyzing the uniaxial stress-strain graphs shown in [33], abdominal skin has a secant stiffness of 1.7 N/mm at a membrane tension reference of 0.136 N/mm, whereas vaginal wall stiffness is 1.45 N/mm at a reference of 0.035 N/mm membrane tension. Rabbits are one model system for mesh performance evaluation. Analysis of the stress-strain curves in [34] yields uniaxial stiffness values for the abdominal wall complex of rabbits of 0.87–0.98 N/mm, whereas [35] report 0.28 N/mm at the same reference membrane tension of 0.136 N/mm. Biaxial stiffness under inflation, however, increases by an order of magnitude to 2.41 N/mm. Similarly, vaginal wall tissue stiffness values at a reference membrane tension of 0.035 N/mm reach from 0.155 N/mm (prolapsed tissue; [29]), 0.675 N/mm (healthy tissue; [33]), 1.4 N/mm (prolapsed tissue; [11]), up to 6.47 N/mm (healthy tissue; [11]).

This variability is due to differences in experimental methodology, *in vivo vs. ex vivo* testing, cadaver testing, animal tissue *vs*. human tissue, pathological *vs*. healthy tissue, as well as the inherent variability of biological soft tissues. This scatter poses a significant problem in determining meaningful mechanical design targets for prosthetic mesh implants and warrants further investigation. Focus should be on the definition of consistent testing procedures based on physiological, *in vivo* loading and stress magnitude conditions.

It has to be noted that the approach of mimicking the ingrown state of the mesh using an elastomer matrix is only partially representative of the *in vivo* condition. This is mainly due to the differences between the embedding procedure and the process of tissue ingrowth. A mesh sample is simply laid into liquid elastomer and the elastomer left to cure. This results in an elastomer-mesh complex that is formed in an unloaded initial configuration, while *in vivo* ingrowth might be expected to happen in a loaded state. Due to this discrepancy, the definition of a secant stiffness between tension values chosen here (pre-force threshold as defined in the experimental protocol and reference membrane tension) might not be representative of the actual *in vivo* load range. This further highlights the need to investigate the expected *in vivo* loading conditions of mesh implants, so to define testing protocols that reproduce physiological states.

When comparing individual meshes in terms of their physical and mechanical parameters, an instructive example can be seen in two meshes manufactured by FEG Textiltechnik: DynaMesh ENDOLAP (DM), used in hernia repair, and DynaMesh PRS (DMPRS), used for pelvic prolapse repair. While their porosity is similar and DM is indeed the heavier of the two, as expected for a hernia mesh, DMPRS is much stiffer in all tested configurations; and up to two orders of magnitude in the case of uniaxial tension.

Similarly, Restorelle (a POP mesh) is the lightest implant with the largest pores; however, its stiffness is clearly above average for most tested configurations. Restorelle is less compliant in the configurations tested here than SPMM, the heaviest, small-pore implant. Note that in previous studies [13,36,37], Restorelle was shown to be more compliant than some of the meshes tested here. This can be attributed to the differences in the experimental protocols. In particular, the present experiments evaluate the response of meshes at a physiological tension level. Thus the low deformation regime determines the measured stiffness in the present work, whereas the ball burst test in [36] depends more on the higher deformation regime.

Lightweight and large porous meshes might be expected to be more compliant than heavy, small porous implants [36]. However, the present analysis shows that while POP meshes are on average indeed lighter and often have larger pores, they are generally stiffer in the physiological loading regime. In these loading configurations, porosity and density alone cannot be predictors for mesh stiffness. Their specific knitting pattern and microstructure can lead to mechanism-like behavior in a physiological loading range, effects that determine their compliance. This calls for a careful evaluation of the mechanical properties of each mesh on several length scales, in conditions representative of those expected *in vivo*.

The present experimental results do not take into account loads in directions other than the main knitting patterns. In fact, the meshes are usually implanted such that their knitting pattern aligns with principal loading directions, such as in line type suspensions (e.g., urethral slings) or sacrocolpopexy procedures. Some meshes, such as DM, Ultrapro (UP) and PE, even have colored filaments interweaved, guiding the physician during implantation. Deviation from this rule might lead to a mechanical response that strongly differs from the data reported here. Similarly, while each mesh was tested in two perpendicular directions, only the stiffer of the two is considered for the present analysis, being indicative of an upper bound of stiffness. Knitted meshes do indeed tend to behave in an anisotropic way, as investigated in [22], with anisotropy indices ranging from 1.0 for SPMM (similar stiffness in both evaluated directions) to 8.0 for DMPRS.

In addition, only one sample per configuration has been tested due to the limited availability of raw mesh material, which also limited the sample size. However, the level of variability for mesh implants reported in the literature [18,22,37,38] is low, justifying the analysis conducted.

## 4. Conclusions

The mechanical biocompatibility of prosthetic mesh implants for hernia and POP repair very likely is an important factor in ensuring their functionality and integration into the host tissue. We see matching mechanical properties in a physiological loading range as desirable and an important step towards reducing clinical complications. 

This study has shown that some meshes designated as suited for POP repair tend to be stiffer than those used for hernia repair, even though the abdominal wall has been shown to be less compliant than the vaginal wall. Additionally, the expectation of lightweight, large pore meshes being more compliant than their counterparts was contradicted by the presented data and specific testing configurations, indicating that a biomechanical analysis of each product is necessary to determine its mechanical suitability. Knowledge of the physiological, *in vivo* mechanical environment in terms of loading configuration and magnitude is required in order to define a suitable design target for optimization of implants. Data reported in the literature show large variations in testing configurations and corresponding stiffness values for pelvic organs and abdominal wall tissue. The consensus for a standardized, physiological mechanical testing procedure is needed for native tissues and implants, opening the path for a conscious mechanical design of prosthetic meshes.
